# Effect of SiO_2_ Content on the Extended Creep Behavior of SiO_2_-Based Wood-Inorganic Composites Derived via the Sol-Gel Process Using the Stepped Isostress Method

**DOI:** 10.3390/polym10040409

**Published:** 2018-04-06

**Authors:** Ke-Chang Hung, Jyh-Horng Wu

**Affiliations:** Department of Forestry, National Chung Hsing University, Taichung 402, Taiwan; d9833004@mail.nchu.edu.tw

**Keywords:** SiO_2_-based wood-inorganic composites, sol-gel process, creep behavior, stepped isostress method, activation volume

## Abstract

In this study, methyltrimethoxysilane (MTMOS) was used as a reagent to prepare SiO_2_-based wood-inorganic composites (WIC_SiO_2__) via the sol-gel process, and subsequently, the extended creep behaviors of WIC_SiO_2__ with weight percent gains (WPGs) of 10%, 20%, and 30% were estimated using the stepped isostress method (SSM). The results revealed that the density of all samples ranged from 426 to 513 kg/m^3^, and no significant difference in the modulus of elasticity (MOE) was noted among all of the samples (10.5–10.7 GPa). However, the MOR of WIC_SiO_2__ with a WPG of 20% (102 MPa) was significantly greater than that of untreated wood (87 MPa). In addition, according to the result using the SSM, the SSM-predicted creep master curve fitted well with the experimental data for the untreated wood and WIC_SiO_2__. This result demonstrated that the SSM could be a useful method to evaluate long-term creep behaviors of wood and WIC_SiO_2__. Furthermore, the activation volume (V*) of the specimens was calculated from the linear slope of Eyring plots, and the resulting V* of all of the WIC_SiO_2__ (0.754–0.842 nm^3^) was lower than that of untreated wood (0.856 nm^3^). On the other hand, the modulus reduction of untreated wood showed 39%, 45%, 48%, and 51% at 5, 15, 30, and 50 years, respectively. In contrast, the modulus reduction of the WIC_SiO_2__ with a WPG of 10% decreased to 25%, 31%, 35%, and 38% at 5, 15, 30, and 50 years, respectively. Similar trends were also observed for other WIC_SiO_2__ with different WPGs. Of these, the WIC_SiO_2__ with a WPG of 20% exhibited the lowest reduction in time-dependent modulus (31%) over a 50-year period. Accordingly, the creep resistance of the wood could be effectively enhanced under the MTMOS treatment.

## 1. Introduction

During the last few years, slicewood or veneer has become increasingly important as an alternative to high quality wood because the supply of large trees continues to diminish and the cost of wood continues to increase [1,2]. However, slicewood or veneer generally have limited exterior applications and long-term utilization because of their low quality [3,4]. Therefore, various wood modifications have been employed to overcome the drawbacks and to introduce multi-functionalities to low-quality wood, such as acetylation, furfurylation, propionylation, heat treatment, and inorganic modification using sol-gel technology [5,6,7,8,9,10]. Among the most suitable methods is inorganic modification using sol-gel technology, and sol-gel-derived wood-inorganic composites (WICs) are considered to have a high potential to obtain value added and improved products [11,12,13,14,15]. The application of metal alkoxides for wood modification using the sol-gel process has been known for more than 20 years [16,17]. During this time, numerous investigations have shown that SiO_2_-based WICs (WIC_SiO_2__) prepared using the sol-gel process have improved flame retardancy, UV and thermal stability, and fungal resistance [6,12,18,19,20,21].

Creep is among the fundamental properties of materials limiting their long-term application as excessive deformation or reduced stiffness occurs over an extended period of time [22]. For material design related to the load-bearing capacity of products, the evaluation of creep behavior is indispensable in engineering applications. However, very little attention has been paid to the creep behavior of WICs. At realistic service-life durations, time is too limited for conducting conventional creep tests, i.e., accelerated tests are necessary [23]. The concept of the accelerated creep test for the prediction of long-term performance is the use of the superposition principle from the combination of exposure time, exposure temperature, and applied load. In other words, a short-term accelerated creep test must be used to obtain the master curve, which is then fitted with an empirical mathematical model. Based on the time-temperature superposition principle (TTSP), the creep behavior of viscoelastic materials can be determined from the stepped temperature in the same manner as time-equivalence [24,25,26]. From this principle, the stepped isothermal method (SIM) has been developed to use stepped increments of temperature for a single sample [27,28,29,30]. Recently, the stepped isostress method (SSM), which can capture the creep behavior of a single sample with a stepwise increase in the stress level, was successfully applied to semi-crystalline thermoplastics and a carbon-fiber-reinforced polymer [23,31,32,33,34]. In comparison to SIM, additionally, the SSM is more advantageous in evaluating creep behavior of wood and WICs, because they are low-thermal-conductivity materials. However, there is little information available on the SSM method characterizing creep deformation and predicting long-term creep behavior of wood and WICs. Therefore, in this study, methyltrimethoxysilane (MTMOS) was used as a reagent to prepare the WIC_SiO_2__, and subsequently, the creep behavior of the WIC_SiO_2__ with different SiO_2_ solid contents was investigated. The time-stress-dependent response and extended creep behavior was evaluated using the SSM. To the best of our knowledge, this is the first work to address the extended creep behavior of wood and WICs using the SSM method.

## 2. Materials and Methods

### 2.1. Materials

Japanese cedar (*Cryptomeria japonica* D. Don) sapwood (20–30 years old) was supplied by the experimental forest of National Taiwan University. The dimensions of the slicewood samples were 3 mm × 12 mm × 58 mm. The oven-dried (o.d.) wood specimens selected for this study were free of defects and exhibited a modulus of elasticity (MOE) of approximately 10.0 GPa. The samples were investigated after extraction using a Soxhlet apparatus for 24 h with a 1:2 (*v*/*v*) mixture of ethanol and toluene, followed by washing with distilled water. The extracted slicewood samples were oven-dried at 105 °C for 12 h, and their weights were measured. The o.d. wood samples were conditioned at 20 °C and 65% relative humidity (RH) for one week prior to the preparation of the WIC_SiO_2__. MTMOS was purchased from Acros Chemical (Geel, Belgium). The other chemicals and solvents used in this experiment were of the highest quality.

### 2.2. Preparation of SiO_2_ Based Wood-Inorganic Composites (WIC_SiO_2__)

The SiO_2_-precursor sol was formulated with MTMOS, methanol, and acetic acid at a molar ratio of 0.12 to 1 to 0.005, 0.04, or 0.08 for preparing the WIC_SiO_2__ with different weight percent gains (WPGs). The wood specimens were impregnated with the prepared sol under reduced pressure (20–28 mmHg) for three days. The impregnated specimens were then placed in an oven at 50 °C for 24 h and 105 °C for another 24 h to age the gels [35]. The WPG was determined based on the o.d. weights.

### 2.3. Determination of Composite Properties

The density of the WIC_SiO_2__ was determined according to the ASTM standard D1037 [36]. The modulus of rupture (MOR) and the modulus of elasticity (MOE) of the specimens were determined using a three-point static bending test at a loading rate of 1.28 mm/min and a span of 48 mm (the specimens with dimensions of 3 mm × 12 mm × 58 mm) according to ASTM standard D790 [37]. Five samples for each WIC_SiO_2__ were conditioned at 20 °C at 65% RH for two weeks prior to testing.

### 2.4. Short-Term Accelerated and Experimental Creep Tests

The short-term stepped isostress method (SSM) was implemented in a universal testing machine (Shimadzu AG-10kNX, Tokyo, Japan) to predict the long-term creep behavior of the WIC_SiO_2__. According to the SSM, the creep strain at a reference temperature is provided by the following equation (Equation (1))

ε(σ_r_, *t*) = ε(σ, *t*/α_σ_) (1)

where ε is the creep strain as a function of stress and time, σ_r_ is the reference stress, σ is the elevated stress, and α_σ_ is the shift factor. Creep tests using the SSM were conducted at a reference stress for 30% of the average breaking load (ABL), and with stepwise increases of stress for 5% ABL. The dwell time was 2 h for each isostress. Additionally, various SSM testing parameters were used to investigate the difference among the different SSM creep tests. The intervals of stress were 5%, 7.5%, 10%, and 12.5% ABL, and the dwell times were 2, 3, and 5 h. 

On the other hand, as shown in Equation (2), the activation volume was calculated based on the Eyring model. This model is more suitable for creep behavior of materials at temperatures below *T*_g_ and was used to estimate the shift factor (α_σ_), which shows the following express rate with the stress level [32,34]

(2) $$\log\alpha_{\sigma }=\log(\frac{\dot{\varepsilon }}{{\dot{\varepsilon }}_{r}})=\frac{V*}{2.303kT}(\sigma -\sigma_{\mathrm{ref}})$$

where $\dot{\varepsilon }$ is the creep rate at the elevated stress (σ), ${\dot{\varepsilon }}_{r}$ is the creep rate at the reference stress (σ_ref_), V* is the activation volume, *k* is Boltzmann’s constant (1.38 × 10^−23^ J/K), and *T* is the absolute temperature.

To validate the master curves derived from the short-term accelerated creep tests, a full-scale experimental creep test was implemented to serve as a basis of comparison. The applied stress was 30% of the ABL, and the mid-span deflection values of the samples were measured and recorded using a linear variable differential transducer (LDVT) for a period of 120 days. Three specimens of each WIC_SiO_2__ were tested. All the samples during the short-term accelerated and experimental creep tests were kept at 20 °C and 65% RH.

### 2.5. Analysis of Variance

All results are expressed as the mean ± SD. The significance of the differences was calculated using Scheffe’s test; *p*-values < 0.05 were considered to be significant.

## 3. Results and Discussion

### 3.1. Mechanical Properties

The density and flexural properties of the WIC_SiO_2__ with different WPGs are listed in Table 1. Accordingly, the density of each composite ranged from 426 to 513 kg/m^3^. In addition, no significant differences in the MOE were found among all the tested samples. The MOE of all the WIC_SiO_2__ (WPGs 10%–30%) is approximately 10.5 GPa, which is basically identical to that of the untreated wood (10.5 GPa). Clearly, the crystallinity of the cellulose greatly influenced the stiffness of the wood [25,38,39]. Thus, this result indicated that the crystallinity of wood was not influenced by the MTMOS treatment. In contrast, the MOR of WIC_SiO_2__ is greater than that of the untreated wood (87 MPa), in particular for the WIC_SiO_2__ with a WPG of 20%. When the WPG of WIC_SiO_2__ reached 10%, 20%, and 30%, the MOR values of the WIC_SiO_2__ were 100, 102, and 101 MPa, respectively. This phenomenon may be attributed to the deposition of silicon compounds on the void of cell walls and intercellular spaces, leading to enhancement of the MOR of the wood.

### 3.2. Creep Master Curves Using the SSM

**Figure 1:**
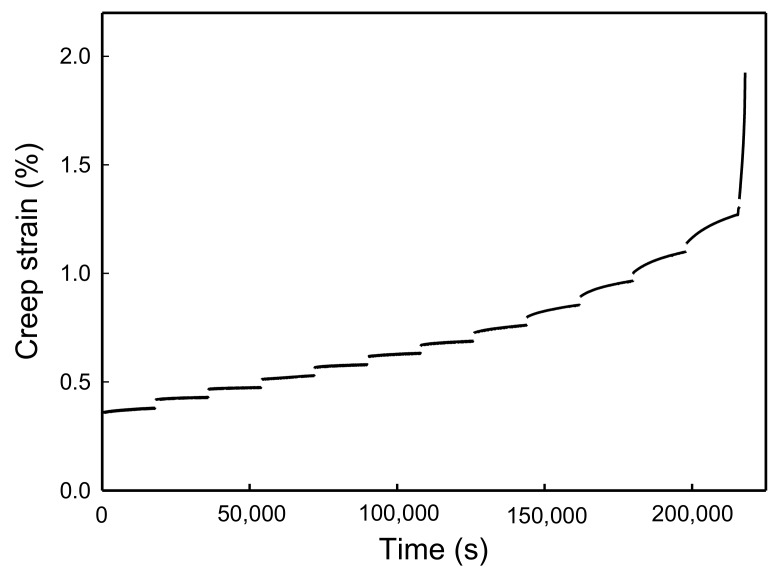
Effect of test stress on creep strain of wood (reference stress: 30% ABL; interval stress: 5% ABL; dwelling time: 5 h).

The key to constructing the creep master curve based on the SSM method is the processing steps of the SSM raw data. Therefore, this section outlines the use of the SSM to predict the long-term creep behavior of wood and the WIC_SiO_2__ from short-term accelerated creep tests at a range of elevated stresses. Using untreated wood as an example, Figure 1 shows the SSM creep curve of wood, which was constructed using the loading sequence of the SSM testing procedure at a reference stress of 30% ABL with a 5% stepwise increase in ABL and a 5 h dwelling time. The master curve of the SSM was constructed via the following four steps of the test raw data: (1) vertical shifting, (2) rescaling, (3) eliminating the period before the onset time, and (4) horizontal shifting.

**Figure 2:**
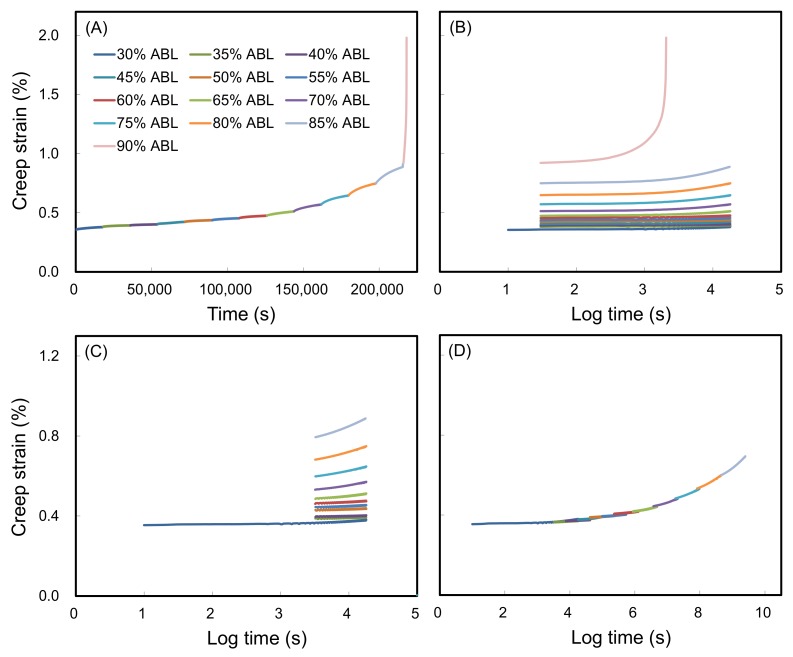
The handling of the test data of the SSM method for wood: (**A**) vertical shifting, (**B**) rescaled creep curves, (**C**) eliminating the onset time of each stress step, and (**D**) horizontal shifting.

Vertical shifting was conducted as the first step in the SSM. An immediate strain jump between the load steps was observed in the SSM creep curve. These jumps are removed by vertical shifting to eliminate the elastic part in the recorded strain, and there was no creep strain at each jump because the wood is elastic under instantaneous strain. Following this shifting process, at each load step, the start of the current curve was linked to the end of the previous curve to generate a continuous creep strain curve as shown in Figure 2A. In addition, the rescaling step of the SSM accounted for deformation and damage during previous steps because of the stress and strain history. In this study, this approach was conducted using a modified method described by Yeo and Hsuan [40]. As shown in Figure 2B, a series of independent creep curves from a stepwise sequential stress increase were shifted to the reference stress level (30% ABL) over a long period of time. As a result of the rescaling, the time before the onset of the creep strain for an individual curve was eliminated as shown in Figure 2C, which is the primary creep region that is most influenced by the stress level and history of the creep strain. After rescaling and eliminating, the individual creep curves are horizontally transited along the time axis to construct the master curve according to the shift factor log($\alpha_{\sigma }$), which is a function of the stress level. Figure 2D illustrates the final smooth master curve of the untreated wood obtained following the SSM processing steps previously described. 

**Figure 3:**
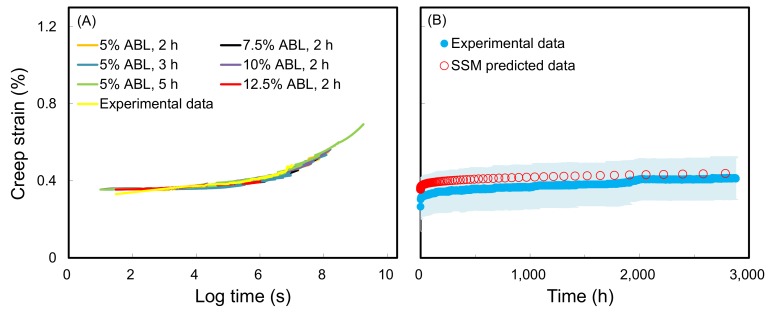
(**A**) Master curves of wood from different SSM testing parameters at a log time scale. (**B**) SSM-predicted creep curve and experimental creep data of wood at a normal time scale. Experimental data are presented as the mean (blue line) ± SD (*n* = 3) (light blue ribbon).

**Figure 4:**
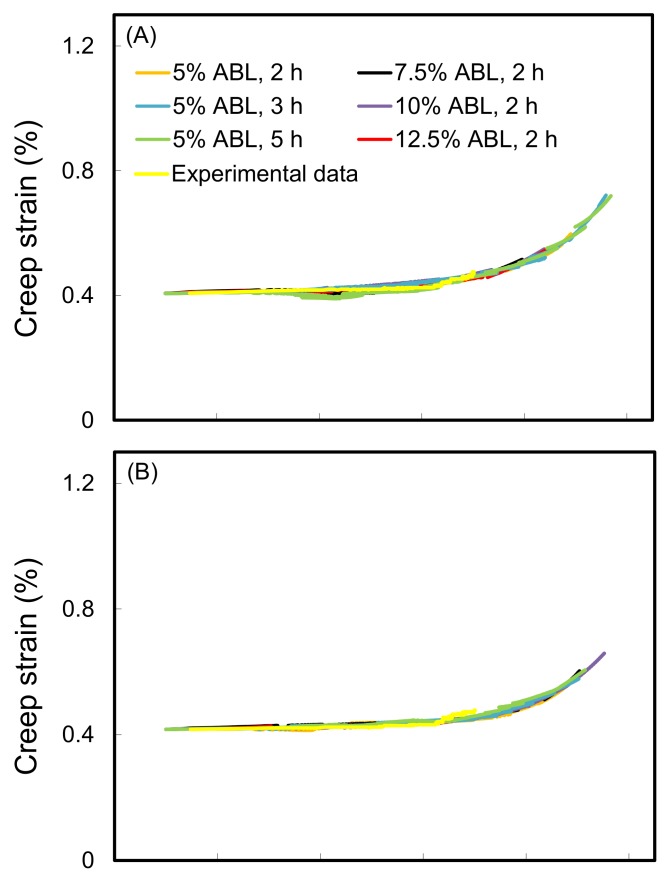

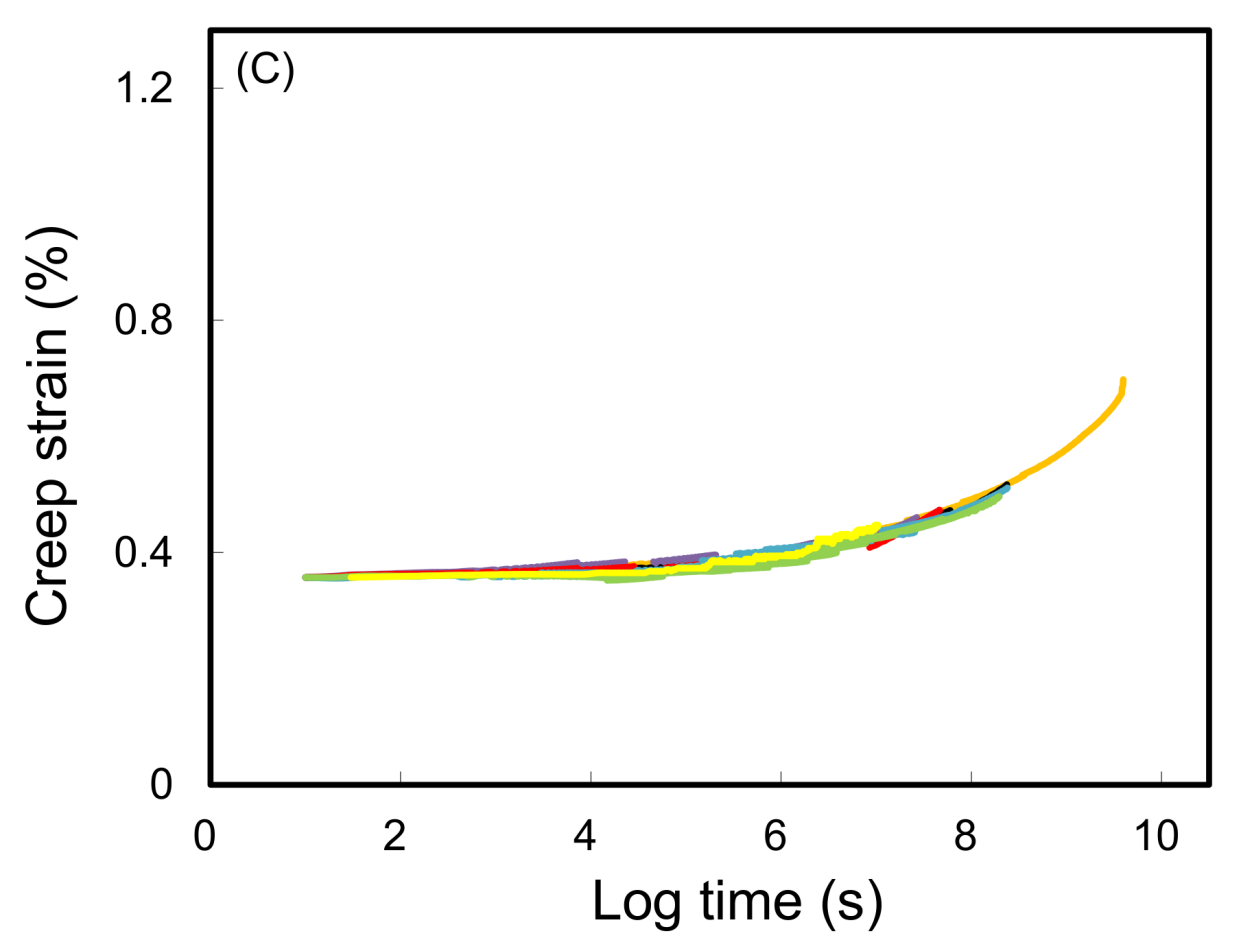
Master curves of WIC_SiO_2__ with WPGs of 10% (**A**), 20% (**B**), and 30% (**C**) using different SSM testing parameters.

On the other hand, the effects of using different stress increments and dwelling time variations on the SSM master curves and the experimental data for untreated wood are shown in Figure 3. The results demonstrated that the master curve was not influenced by the test conditions for a given wood sample (Figure 3A), and the curves were highly consistent with the experimental data. Similarly, the resulting master curves of all the WIC_SiO_2__ were highly consistent with their experimental data (Figure 4). These results showed that the predicted creep curves of the wood and WIC_SiO_2__ using the SSM procedure were valid in matching long-term creep behavior. A similar result was reported by Hadid et al. [34].

**Figure 5:**
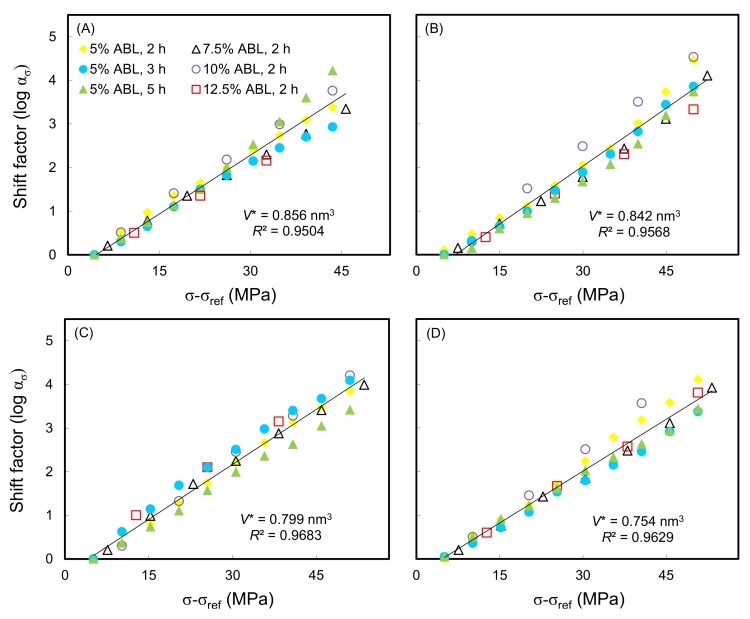
Typical Eyring equation plots of wood (**A**) and WIC_SiO_2__ with WPGs of 10% (**B**), 20% (**C**), and 30% (**D**) at a reference load of 30% ABL.

Additionally, for a given set of test conditions, the data corresponding to the sample that failed during the jump to the final load step after a certain time were ignored. Meanwhile, Figure 5 shows the relationship between the shift factor and stress level. It can be seen that a linear regression was performed to determine the slope of the plot of the shift factor versus the stress level, as validated by the values of the coefficient of determination (*R*^2^) being > 0.95. This result indicated that the superposition method used in conjunction with the SSM was validated to generate the creep master curve, indicating that the same creep mechanism is performed for each load sequence. The activation volume (V*) was calculated from the linear slope of the Eyring plot (Equation (2)). Accordingly, the V* of the WIC_SiO_2__ (0.754–0.842 nm^3^) was markedly lower than that of the untreated wood (0.856 nm^3^), and the value decreased with the increasing WPG of the WIC_SiO_2__. For a chemical process, the V* is interpreted as the difference between the partial molar volumes of the transition state and the sums of the partial volumes of the reactants at the same temperature and pressure according to transition state theory [41]. However, Giannopoulos and Burgoyne [32] reported that the V* may be consistent with the idea that the molecules are pulled apart during creep. Thus, this result implied that the MTMOS treatment limited the pull volume of molecules during the creep period of wood.

### 3.3. SSM-Predicted Creep Curves

**Figure 6:**
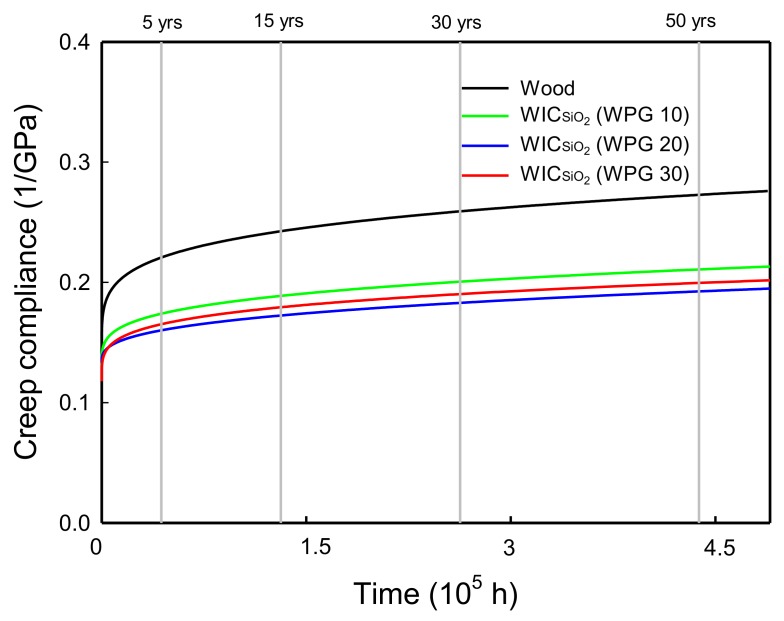
SSM predicted creep data of wood and WIC_SiO_2__ with different WPGs.

The SSM-predicted compliance master curves of wood and WIC_SiO_2__ with different WPGs on a normal time scale are shown in Figure 6. The master curves were modeled using the Findley power law [42], which is presented in the equation

*S*(*t*) = *S*_0_ + *at^b^* (3)

where, *S*(*t*) is the time-dependent compliance value, *S*_0_ is the instantaneous elastic compliance value, *a* and *b* are constant values, and *t* is the elapsed time. As listed in Table 2, the model fits the SSM master curves for wood and WIC_SiO_2__ very well; all of the *R*^2^ values of the model were greater than 0.994. Additionally, all of the creep compliances of the WIC_SiO_2__ were less than those of the untreated wood during the creep duration, and the compliance of the WIC_SiO_2__ with a WPG of 20% was the lowest.

On the other hand, the instantaneous elastic compliances (*S*_0_) and the predicted time-dependent compliances (*S*(*t*)) of all the samples over the 5–50-year periods are listed in Table 2. Among the equation parameters, the WIC_SiO_2__ with different WPGs have lower *S*_0_ values (0.117–0.133 GPa^−1^) compared to that of untreated wood (0.134 GPa^−1^). For the predicted compliance, the untreated wood showed 0.22, 0.24, 0.26, and 0.27 GPa^−1^ at 5, 15, 30, and 50 years, respectively. Following MTMOS treatment, the compliance values of the WIC_SiO_2__ with a WPG of 10% significantly decreased to 0.17, 0.19, 0.20, and 0.21 GPa^−1^ at 5, 15, 30, and 50 years, respectively. Similar trends were also observed for the WIC_SiO_2__ with WPGs of 20% and 30%, but their compliances decreased with increasing WPGs up to 20%. These results implied that the MTMOS treatment improved the creep resistance of wood because of the deposition of silicon compounds on the void of cell walls and intercellular spaces. These findings are similar to the flexural properties data.

Furthermore, to estimate the creep resistance of a sample under long-term conditions, the modulus reduction was calculated using the equation (Equation (4))

(4) $$\mathrm{Modulus}\;\mathrm{reduction}(\%)=[1-\frac{S_{0}}{S\left(t\right)}]\times 100$$

As listed in Table 2, the modulus of the untreated wood would decrease by 51% over 50 years. However, the modulus reduction of all WIC_SiO_2__ significantly decreased in a range of from 31%–41% over a 50-year period. Of these, the smallest modulus reduction was found for WIC_SiO_2__ with a WPG of 20% (31%). Accordingly, these results demonstrated that the creep resistance of the wood would be improved under the MTMOS treatment.

## 4. Conclusions

Japanese cedar slicewood was used to prepare WIC_SiO_2__ using the sol-gel process, and the extended creep behavior of the specimens was estimated using the stepped isostress method (SSM). The results showed that the incorporation of SiO_2_ into the void of cell walls and intercellular spaces increased the MOR of wood. In addition, the SSM was suitable for constructing the master curve of wood and WIC_SiO_2__. Accordingly, the creep compliance of WIC_SiO_2__ was less than that of the untreated wood during the creep duration. Meanwhile, the modulus reduction of the untreated wood was 51% at 50 years, but the reduction value decreased following MTMOS treatment. Among all the WIC_SiO_2__, the WIC_SiO_2__ with a WPG of 20% exhibited the lowest reduction in time-dependent modulus (31%) over a 50-year period. In addition, the activation volume was 0.856, 0.842, 0.799, and 0.754 nm^3^ for wood and WIC_SiO_2__ with WPGs of 10%, 20%, and 30%, respectively. Therefore, the incorporation of SiO_2_ into the wood using the sol-gel process could improve its creep resistance, particularly with a WPG of 20%. The results of this study provide a reliable SSM approach for predicting the long-term creep behavior of wood and WIC_SiO_2__.

## Figures and Tables

**Table 1:** Effects of SiO_2_ content on the flexural properties of various WIC_SiO_2__.

Specimen	WPG (%)	Density (kg/m^3^)	Flexural properties	
			MOE (GPa)	MOR (MPa)
Wood	‒	426 ± 31 ^b^	10.5 ± 1.5 ^a^	87 ± 8 ^b^
WIC_SiO_2__	10	513 ± 22 ^a^	10.7 ± 0.4 ^a^	100 ± 4 ^ab^
	20	438 ± 16 ^b^	10.5 ± 1.1 ^a^	102 ± 11 ^a^
	30	459 ± 37 ^b^	10.5 ± 0.3 ^a^	101 ± 4 ^ab^

Values are the mean ± SD (*n* = 5). Different letters within a column indicate significant differences (*p* < 0.05).

**Table 2:** Predicted creep compliances of wood and WIC_SiO_2__ with different WPGs.

Specimen	WPG (%)	*S*_0_ (GPa^−1^)	*a*	*b*	*R*^2^	*S*(*t*) (GPa^−1^)				Modulus reduction (%)			
						Time (Years)				Time (Years)			
						5	15	30	50	5	15	30	50
Wood	‒	0.134	0.0097	0.21	0.9963	0.22	0.24	0.26	0.27	39	45	48	51
WIC_SiO_2__	10	0.130	0.0027	0.26	0.9947	0.17	0.19	0.20	0.21	25	31	35	38
	20	0.133	0.0007	0.34	0.9995	0.16	0.17	0.18	0.19	17	23	27	31
	30	0.117	0.0040	0.23	0.9981	0.17	0.18	0.19	0.20	29	35	39	41

*S*(*t*) = *S*_0_ + *at^b^*, where *S*(*t*) is the time-dependent compliance value, *S*_0_ is the instantaneous elastic compliance value, and *a* and *b* are constant values.
